# Effect of Calcium Oxide on the Mechanical and Structural Properties of Metakaolin-Based One-Part Geopolymer

**DOI:** 10.3390/molecules31173132

**Published:** 2026-09-07

**Authors:** Shiqiang Sun, Weijie Meng, Zeyuan Lv, Yufang Zhai

**Affiliations:** 1 School of Physics and New Energy, Xuzhou University of Technology, Xuzhou 221111, China; 2 School of Chemical Engineering and Technology, China University of Mining and Technology, Xuzhou 221116, China; 3 Jining Institute of Industrial Technology, Jining 272073, China

**Keywords:** geopolymer, calcium oxide, compressive strength, microstructure

## Abstract

One-part geopolymer has emerged as a promising alternative to ordinary Portland cement. In this study, the effect of CaO dosage on the compressive strength of one-part geopolymers was systematically investigated, and its underlying modification mechanism was revealed via multi-scale characterizations including XRD, FTIR, TG, NMR and nitrogen adsorption–desorption. The results show that the compressive strength of the samples at all curing ages exhibits a trend of sharp initial decrease, followed by a slight rebound, and then a secondary decline with the increase in CaO dosage. All CaO-containing specimens exhibit significantly lower strengths than the CaO-free reference. Specifically, the reference sample achieves the highest 28-day compressive strength of 56.6 MPa. The strength of the sample at each curing age drops to the minimum at 5% CaO dosage, with a 28-day strength of only 17.9 MPa. Partial strength recovery of the sample is achieved at 7.5% CaO dosage. The strength deterioration is mainly attributed to the rapid hydration of CaO, which consumes free water and reactive silicon and hinders the generation of N-A-S-H gel rather than directly disrupting the aluminosilicate network. Meanwhile, the hydration products are continuously carbonated to form calcium carbonate, and the carbonation-induced volume expansion at excessive dosage may induce microcracks in the matrix that impair the structural integrity. Only at a moderate dosage of 7.5% CaO can a slight strength rebound be realized through the possible formation of C-S-H-type phases and the pore-filling effect. This study provides a theoretical basis for the material design and performance regulation of one-part geopolymers.

## 1. Introduction

The production of ordinary Portland cement is accompanied by substantial carbon dioxide emissions, making it a major contributor to global warming [1]. According to literature reports, approximately 0.66–0.82 tons of CO_2_ are emitted per ton of ordinary Portland cement manufactured, and the cement industry accounts for 5–7% of global anthropogenic CO_2_ emissions [2,3]. In response to increasing environmental pressures, the development of low-carbon cementitious materials has become a research priority in both academia and industry. Geopolymer, a class of inorganic polymers with a three-dimensional network structure composed of silicon-oxygen and aluminum-oxygen tetrahedra linked by bridging oxygens, consumes only one-third to one-half of the energy required for ordinary Portland cement production and can reduce carbon emissions by more than 60% [4,5]. Moreover, geopolymer exhibits excellent mechanical properties, superior corrosion resistance, and good thermal stability, making it one of the most promising alternatives to ordinary Portland cement [6,7].

Geopolymer is typically prepared by reacting an aluminosilicate precursor with highly caustic and viscous liquid alkali activators (e.g., NaOH or sodium silicate solutions) [8,9]. However, the storage, transportation, and handling of liquid alkalis pose safety risks, and their high viscosity increases construction difficulty, thereby limiting their large-scale application in cast-in situ projects [10]. To overcome this limitation, one-part geopolymer emerged. In this case, a solid activator is pre-mixed with the aluminosilicate precursor, and only water is added before use, significantly improving construction convenience and field applicability [11]. Elzeadani et al. [10] summarized the raw material selection, mechanical properties, and durability characteristics of one-part materials, concluding that they hold great promise for in situ applications.

The mechanical performance of one-part geopolymer is strongly dependent on the chemical composition of the raw materials, the Si/Al ratio, and the type and dosage of additives [12]. The addition of appropriate alkaline oxides can effectively regulate the geopolymerization process, optimize the pore structure, and improve the composition of the binding gels [13]. In our previous work, we systematically investigated the effect of MgO on the compressive strength of metakaolin-based one-part geopolymers and found that an optimal MgO content led to the formation of a composite structure comprising magnesium silicate hydrate (M-S-H) and N-A-S-H gels, increasing the 28-day strength to 68.94 MPa [14]. CaO is also a widely available and low-cost alkaline oxide. Its hydration reaction rapidly produces Ca(OH)_2_, which releases OH^−^ ions and creates a strongly alkaline local environment for geopolymerization [15]. However, the rapid hydration of CaO is accompanied by significant heat release, and CaO is prone to carbonation to form CaCO_3_, which may affect the stability of the gel phases. Moreover, excess Ca^2+^ may interfere with the formation of the N-A-S-H network [16].

Several researchers investigated the role of CaO in alkali-activated materials, but most studies focused on slag-based systems. Temuujin et al. [17] systematically compared the effects of different calcium compounds on the mechanical properties of fly ash-based geopolymers and found that calcium oxide exhibited significantly better modification effects than calcium carbonate and calcium sulfate. With a CaO dosage of 10%, the 7-day compressive strength of the fly ash-based geopolymer increased by 65%. Kim et al. [18] explored the mechanical strength difference between cementitious materials of ground granulated blast furnace slag activated by CaO and Ca(OH)_2_ and found that CaO was more effective as an activator than Ca(OH)_2_. Cai et al. [19] compared the mechanical properties of CaO- and MgO-activated slag-solidified soils and found that the CaO system exhibited higher unconfined compressive strength after 7 days of curing, while the MgO system showed better long-term mechanical performance. In metakaolin-based systems, Chen et al. [20] systematically investigated the influence of calcium content on geopolymer gels and found that, with the increase in calcium content, the mechanical properties of geopolymers decreased with a threshold effect. The calcium-containing systems mainly generated N(C)-A-S-H amorphous phase and calcite.

However, the aforementioned studies on CaO-modified geopolymers have predominantly employed liquid alkali activation. The role of CaO in one-part metakaolin-based geopolymers and its influence on mechanical properties remain insufficiently understood. Based on the above literature, the following research hypothesis was proposed: CaO may exert dual competing effects on metakaolin-based one-part geopolymers—promoting C-(A)-S-H gel formation at moderate dosages while suppressing N-A-S-H gel and inducing carbonation-related deterioration at excessive levels. Therefore, in this study, a combination of characterizations including XRD, FTIR, TG, NMR, and pore structure analysis was employed to systematically evaluate the compressive strength evolution and solid-phase microstructural characteristics of one-part geopolymers with various calcium dosages, thereby elucidating the underlying mechanism of calcium oxide in the metakaolin-based one-part geopolymer system.

## 2. Results and Discussion

### 2.1. Compressive Strength of Geopolymers

**Figure 1:**
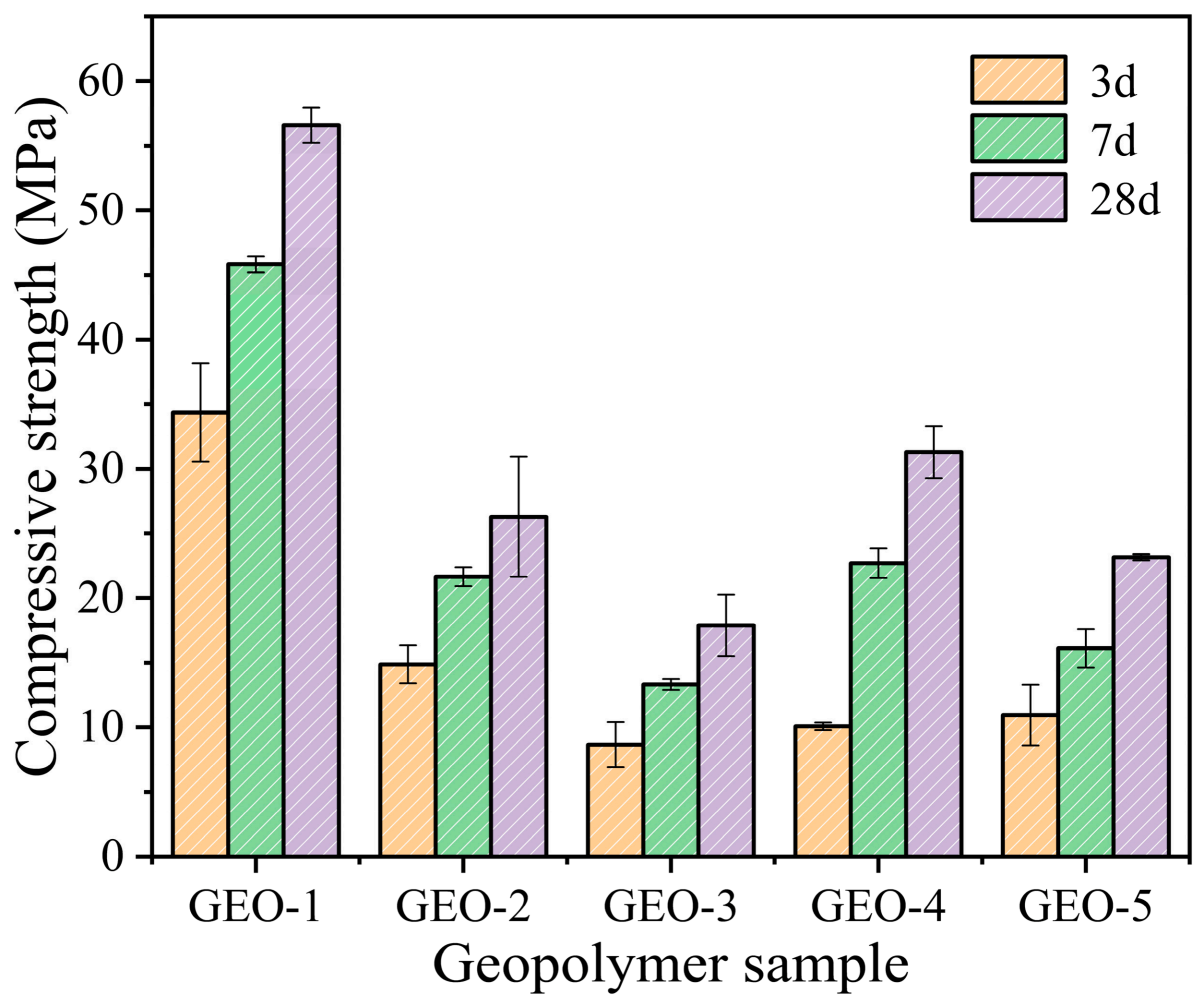
Box-and-whisker plot of compressive strength for geopolymer samples with different CaO dosages at 3, 7, and 28 days. The box indicates the interquartile range (25th–75th percentile); the horizontal line denotes the median; the whiskers extend to the minimum and maximum values within 1.5× IQR; points beyond the whiskers represent outliers.

Figure 1 depicts the evolution of compressive strength for metakaolin-based one-part geopolymers with varying CaO dosages after curing for 3, 7 and 28 d. For all curing ages, the compressive strength of the geopolymers exhibited a trend of a sharp initial decrease, followed by a slight rebound as the CaO content increased. Notably, every CaO-incorporated sample exhibited lower mechanical performance than the reference sample without CaO. The CaO-free sample (GEO-1) achieved the highest compressive strengths of approximately 34, 45, and 56 MPa at 3, 7, and 28 days, respectively. When the CaO dosage reached 5% (GEO-3), the strength reached its minimum at all curing ages, falling to approximately 8, 13, and 17 MPa. A noticeable strength increase was observed when the CaO dosage was further elevated to 7.5% (GEO-4), with its 28 d compressive strength rebounding to 31.3 MPa. As the CaO dosage reached 10% (GEO-5), the compressive strength exhibited a minor secondary decrease to 23.2 MPa. These results indicated that the introduction of CaO has an overall detrimental effect on the mechanical properties of geopolymers, with a particularly stronger inhibitory effect on early-age compressive strength. This behavior was markedly different from the strength-enhancing effect observed with an appropriate amount of MgO in our previous study [14].

It should be noted that the liquid-to-solid ratio was not kept constant but was increased progressively from 0.400 to 0.500 with increasing CaO content. This adjustment was necessary because CaO has a high water demand due to its rapid hydration; without sufficient water, the paste cannot form a workable slurry, and the reaction cannot proceed properly. Therefore, the variation in water content is a consequence of CaO addition rather than an independent experimental variable. In the relatively narrow range of 0.400 to 0.500, the influence of water content on strength is expected to be secondary compared to the effect of CaO. This is supported by the fact that the compressive strength across all curing ages follows a consistent non-monotonic trend: it decreases sharply at 5% CaO, recovers at 7.5% CaO, and declines again at 10% CaO. This trend correlates directly with CaO dosage rather than with the monotonically increasing water content.

### 2.2. Solid Analysis

**Figure 2:**
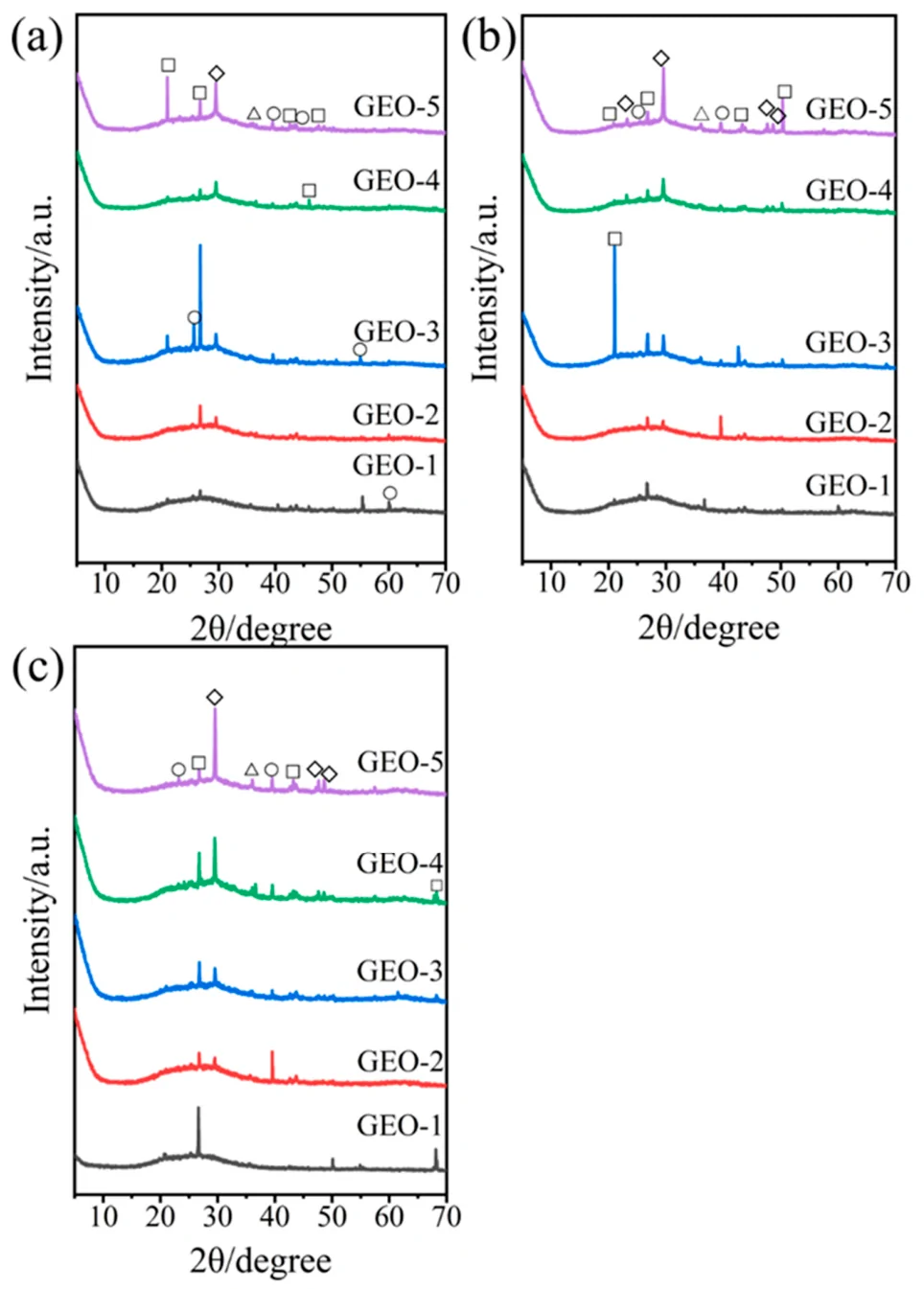
XRD patterns of all geopolymer samples at different curing times: (**a**) 3 d, (**b**) 7 d, (**c**) 28 d. The three subplots are vertically arranged with identical 2θ scales to facilitate direct comparison of peak positions across curing ages and CaO levels. Symbols: ☐, Quartz (SiO_2_); ○, Anatase (TiO_2_); ◇, Calcite (CaCO_3_); △, Hematite (Fe_2_O_3_).

Figure 2 shows the XRD patterns of one-part geopolymers at different curing ages. Characteristic peaks of quartz (SiO_2_) derived from metakaolin and the solid activator, as well as residual anatase (TiO_2_) peaks from metakaolin, were observed in all samples. The broad diffuse peak in the range of 20°~30° was the characteristic diffraction peak of the amorphous geopolymer gel phase, indicating the formation of a cementitious structure dominated by aluminosilicate networks [21]. In addition, weak characteristic peaks of hematite (Fe_2_O_3_), originating from impurities in the raw materials, were detected in some samples. Compared with the CaO-free sample GEO-1, all CaO-containing specimens exhibited a distinct calcite (CaCO_3_) peak at 2θ = 29.4°, while no free CaO diffraction peak was detected, indicating that CaO had been completely hydrated to Ca(OH)_2_ at an early stage, and part of the Ca(OH)_2_ had subsequently undergone carbonation. With the extension of curing age, the intensity of CaCO_3_ characteristic peaks increased gradually, while the intensity of the broad peak of the amorphous gel phase showed an overall decreasing trend.

**Figure 3:**
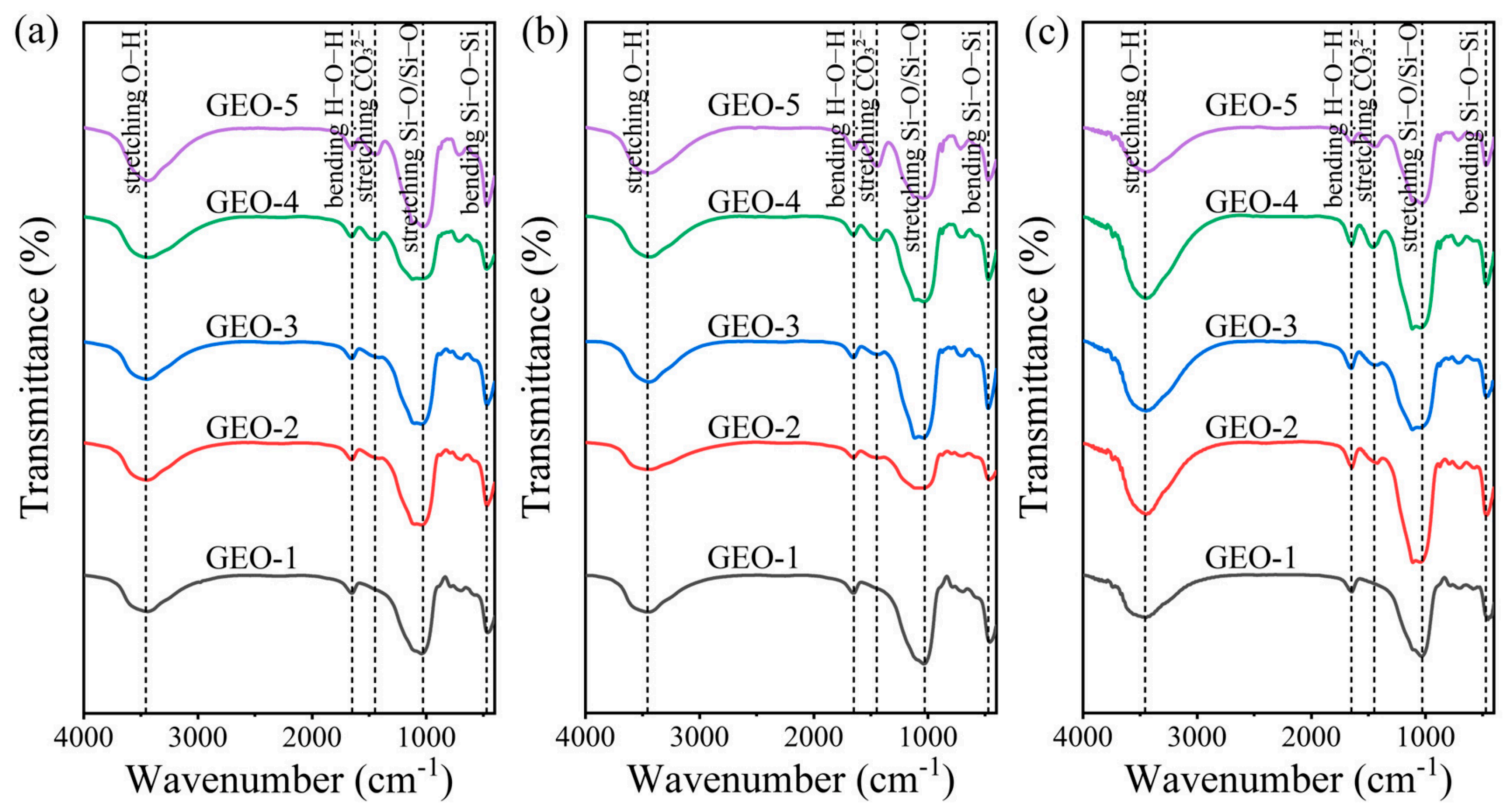
FTIR spectra of different samples at different curing times: (**a**) 3 d (**b**) 7 d (**c**) 28 d.

Figure 3 presents the FTIR spectra of geopolymers at different curing ages. Typical characteristic peaks of geopolymers were observed in all samples. The broad peak at 3400~3500 cm^−1^ corresponded to the stretching vibration of hydroxyl groups, and the peak at 1630~1650 cm^−1^ was attributed to the bending vibration of water molecules, both of which reflected the content of bound water in the system [22]. The strong absorption peak in the range of 1000~1120 cm^−1^ was the asymmetric stretching vibration of Si-O-T (T = Si or Al) bonds [23], among which the peak at ~1025 cm^−1^ corresponded to the Si-O-Al bonds in the N-A-S-H gel and the peak at ~1110 cm^−1^ corresponded to the Si-O-Si bonds in unreacted aluminosilicate raw materials [24,25]. The peak near 450 cm^−1^ corresponded to the bending vibration of Si-O-Si bonds [26]. In addition, all CaO-containing samples showed an obvious stretching vibration peak of carbonate around 1430 cm^−1^ [27], which was consistent with the calcite phase detected by XRD.

**Figure 4:**
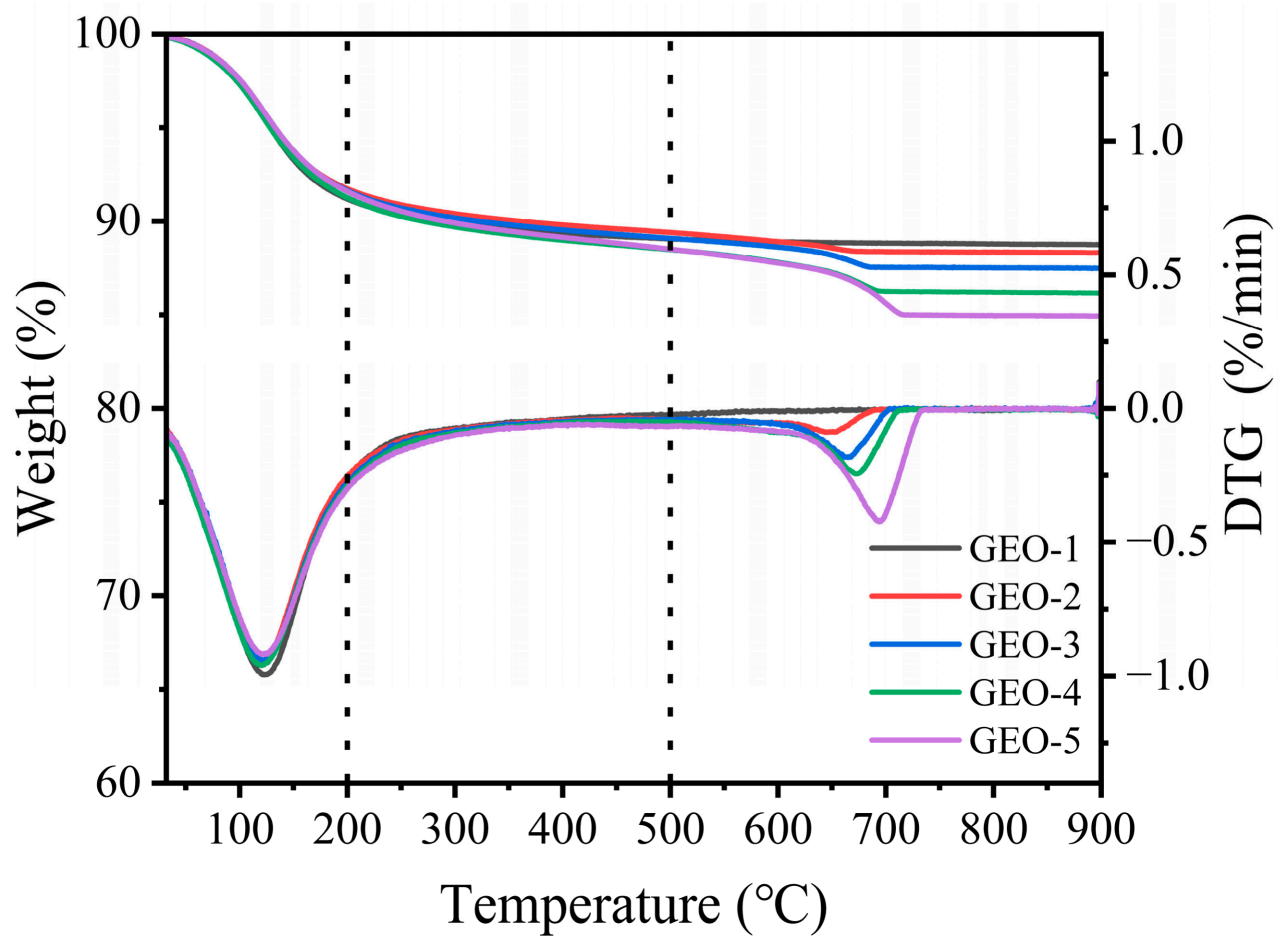
TG–DTG curves of geopolymer samples cured for 28 d.

Figure 4 presents the TG–DTG curves of geopolymer samples cured for 28 d. All samples exhibited similar three-step thermal weight loss characteristics. The first stage ranged from room temperature to 200 °C, which mainly corresponded to the removal of physically adsorbed water on the sample surface and weakly bound capillary water within the gel pores. The DTG curves showed a sharp and symmetric weight loss peak around 100 °C, and the thermal weight loss rate of all samples in this stage was approximately 10%. This was attributed to the fact that the rapid hydration of CaO to form Ca(OH)_2_ contributed a large amount of bound water, which masked the influence of different degrees of geopolymerization on the weight loss in this stage. The second stage was from 200 to 500 °C, which corresponded to the condensation reaction of Si-OH and Al-OH groups in the geopolymer gel, leading to the continuous removal of structural water and the formation of Si-O-T (T = Si or Al) tetrahedral structures [28]. In this stage, the DTG curves of all samples overlapped considerably, indicating that the addition of CaO did not introduce additional thermal reactions. The third stage was from 500 to 750 °C, which corresponded to the thermal decomposition of CaCO_3_ in the system, with a distinct decomposition peak appearing around 700 °C on the DTG curves. With the increase in CaO dosage, the decreasing amplitude of the TG curve in this interval increased continuously, and the intensity and integrated area of the DTG peak also increased synchronously. This indicated that a higher CaO dosage led to a greater amount of calcite formed by carbonation of Ca(OH)_2_ in the system, and consequently, the total thermal weight loss of the samples gradually increased with rising CaO content.

### 2.3. Mechanism Analysis

**Figure 5:**
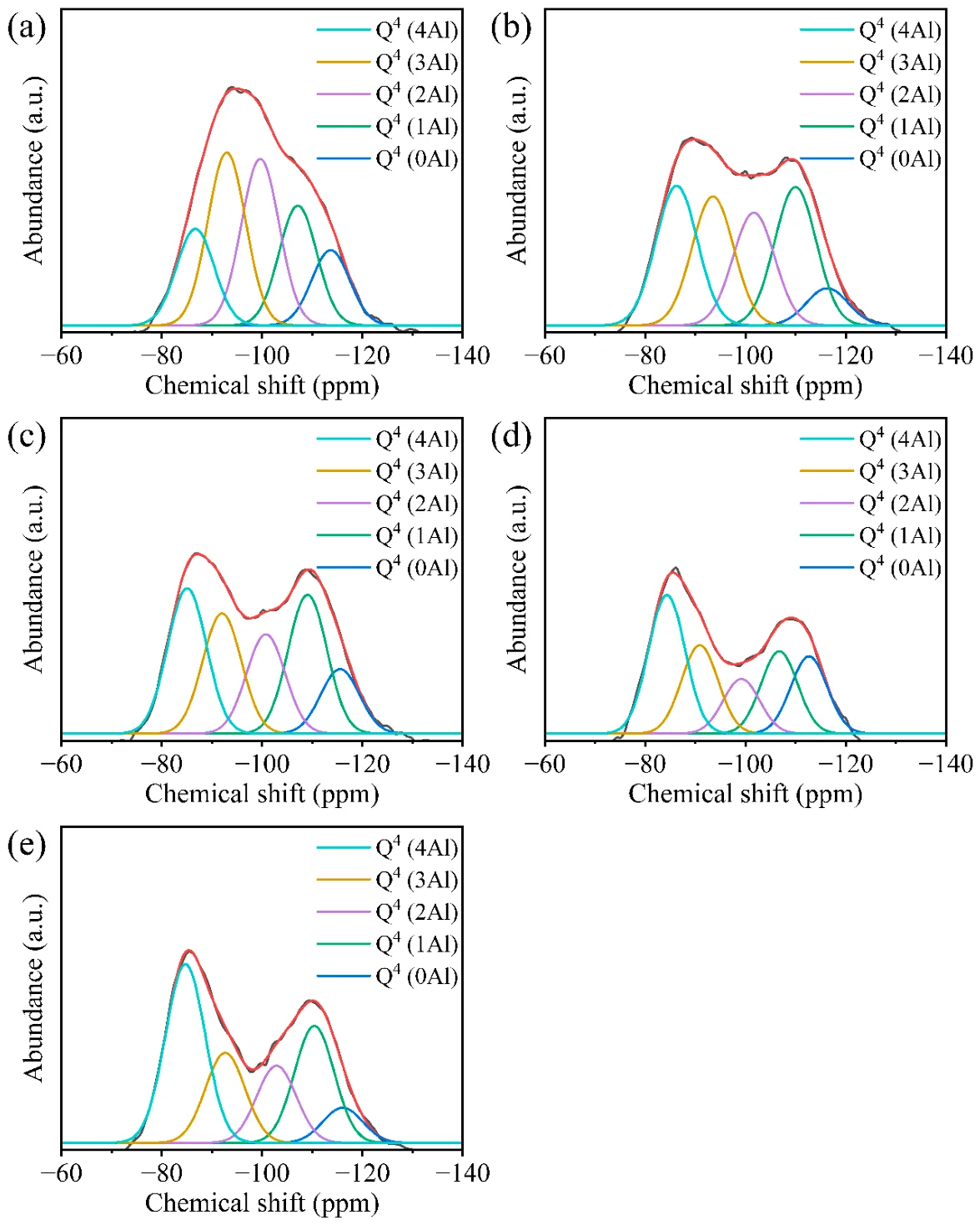
^29^Si magic-angle spinning NMR spectra of the geopolymer samples after 28 days of curing: (**a**) GEO-1; (**b**) GEO-2; (**c**) GEO-3; (**d**) GEO-4; (**e**) GEO-5.

**Figure 6:**
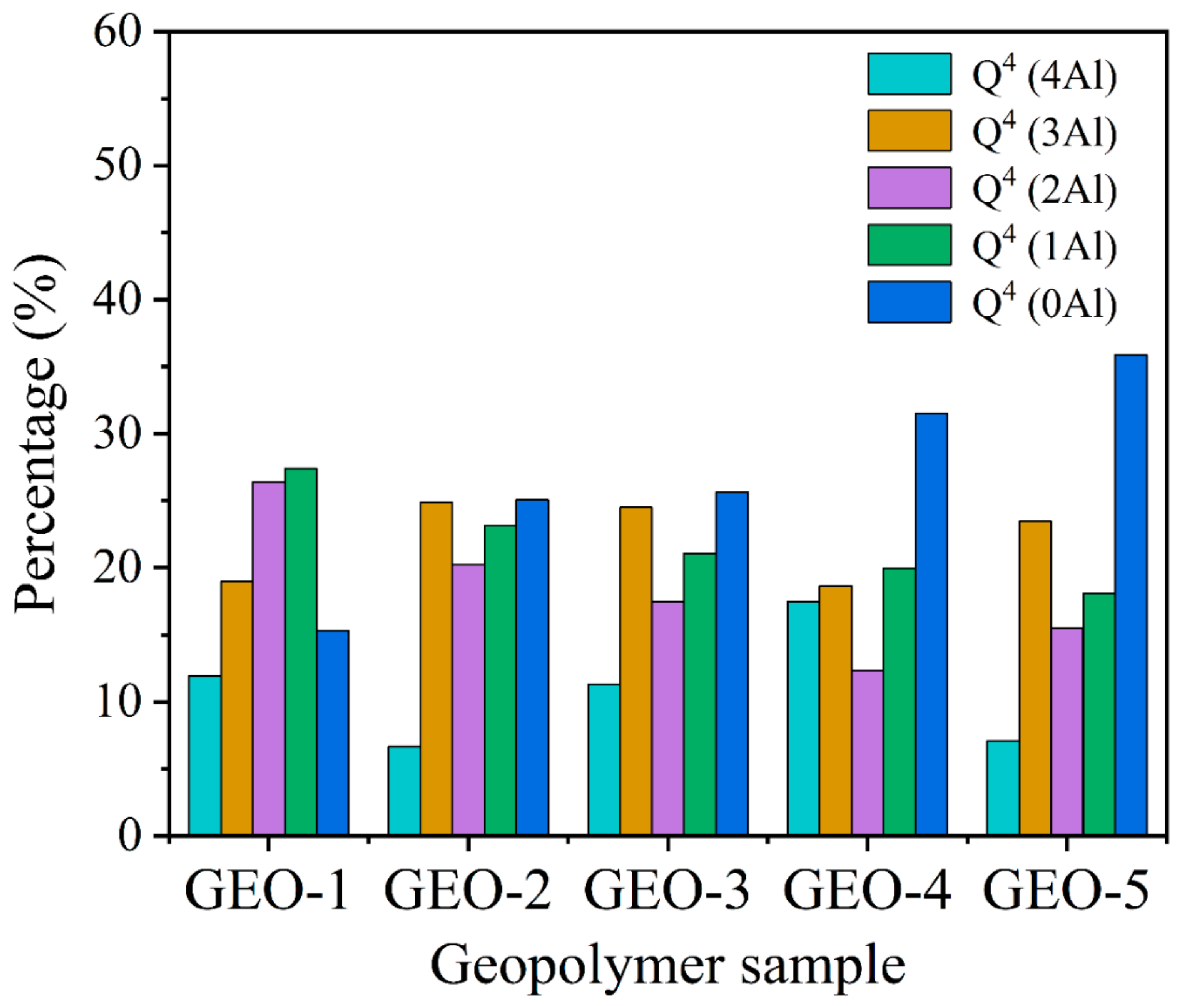
Normalized summary of the Si coordination environments within the geopolymer samples cured for 28 d, as determined from deconvolution of the ^29^Si NMR spectra.

Based on the above results, it can be found that the addition of CaO has a negative effect on the preparation of one-part geopolymers. In order to elucidate the structural consequence led by CaO, solid-state ^29^Si NMR was conducted. Figure 5 displays the original ^29^Si NMR spectra and their gaussian deconvolution outcomes of geopolymer samples with different CaO dosages after 28 days curing. The resonance peaks located at −82 to −90 ppm, −85 to −94 ppm, −92 to −98 ppm, −96 to −107 ppm, and −102 to −115 ppm correspond to Q^4^(4Al)/Q^2^, Q^4^(3Al)/Q^3^, Q^4^(2Al), Q^4^(1Al), and Q^4^(0Al), respectively [29,30]. The relative contents of each structural unit are summarized in Figure 6. For the sample GEO-1, the broad resonance peak of the Al-rich N-A-S-H gel phase at −90 ppm was overwhelmingly dominant, and the broad peak of the unreacted silica fume quartz phase at −108 ppm was only a faint shoulder peak. This demonstrated that the pure metakaolin system undergoes complete geopolymerization, forming an aluminosilicate gel network with uniform composition and appropriate polymerization degree [27]. As the CaO dosage increased, the broad gel-phase peak at −90 ppm shifted notably to the left. This leftward shift indicated that the average number of aluminum atoms in the coordination environment of silicon decreased gradually with increasing CaO content, corresponding to a transition from high-aluminum coordination to low-aluminum coordination. This occurred because Ca^2+^ had a stronger affinity for silicate ions than for Al^3+^, causing aluminum to detach from the silicon coordination shell [31]. Concurrently, the overall intensity of the broad peak decreased continuously with increasing CaO dosage, being highest in GEO-1 and weakest in GEO-3, which directly reflected the reduction in the total amount of aluminum-associated silicon species in the gel. Additionally, strong resonance signal peaks at −108 ppm were observed in samples Geo-2 to Geo-5. Combined with the summary chart of relative content, it can be seen that, as the calcium oxide content increases, the proportion of Q^4^(0Al) gradually rises, which to some extent indicates a reduction in the geopolymerization reaction.

To further explore the role of calcium in the geopolymers, the reasons for the variation in compressive strength of geopolymer samples with different calcium oxide dosages were analyzed. The added calcium oxide dissolves and releases calcium ions; Ca^2+^ has a stronger affinity for binding with active silicate ions than with Al^3+^, thus it preferentially consumes the reactive silica components that would otherwise be used to form the N-A-S-H gel. Combined with the XRD patterns, it was found that the only calcium-related phase in the geopolymer is calcium carbonate after 3 and 7 days of curing. Therefore, calcium ions may have combined with silicate ions to form amorphous substances of C-S-H or C-A-S-H gels.

CaO preferentially undergoes hydration and cannot directly participate in the geopolymerization network. This is because the hydration of CaO is kinetically much faster than the dissolution of metakaolin in alkaline media. Upon contact with water, CaO rapidly forms Ca(OH)_2_, with significant heat release. The subsequent release of Ca^2+^ and its reaction with silicate species are secondary processes triggered by hydration, rather than direct reactions of CaO itself. On this basis, the variation in compressive strength with different CaO dosages can be further elucidated from the perspective of reaction processes and structural evolution. It should be noted that the liquid-to-solid ratio was not constant but increased gradually with increasing CaO content. Nevertheless, a consistent trend in compressive strength was observed at curing ages of 3, 7, and 28 days, suggesting that the evolution of gel structure is primarily governed by CaO content, while the variation in water content plays a secondary role. For GEO-2 and GEO-3 samples, the hydration-driven processes impose dual influences on system performance. On the one hand, this process consumes a large amount of free water, reducing the available reaction medium for aluminosilicate dissolution. The rapid formation of Ca(OH)_2_ also increases the local ionic strength, which may hinder the dissolution and depolymerization of metakaolin. Meanwhile, the subsequently formed Ca(OH)_2_ is prone to carbonation, which consumes OH^-^ and reduces the effective alkalinity of the system over time. On the other hand, the released Ca^2+^ preferentially combines with dissolved silicate species to form initial C-S-H gel precursors, reducing the amount of active silica available for constructing the N-A-S-H network. At this stage, even if the amount of added water is increased, it cannot effectively compensate for the loss of water and alkalinity caused by CaO hydration. As a result, the formation of N-A-S-H gel is significantly reduced, the gel network becomes discontinuous, and the structure is dominated by loose packing, leading to a notable decrease in compressive strength [32]. These observations correspond to the NMR results, where the content of Q^4^(2Al) decreases and that of Q^4^(0Al) increases, indicating a weakening of the geopolymerization reaction.

When the CaO dosage increases to 7.5% (GEO-4), a certain degree of compensatory effect begins to emerge in the system. On the one hand, the relatively higher liquid-to-solid ratio partially alleviates the consumption of water during CaO hydration, thereby maintaining a sufficient reaction medium for subsequent processes. On the other hand, as the reaction proceeds, the initially formed Ca(OH)_2_ may further participate in the aluminosilicate system, reacting with dissolved silicate and aluminate species to form C-(A)-S-H-type phases, as indirectly suggested by the FTIR and NMR results. In contrast to the dispersed C-S-H precursors formed at lower CaO dosages, the gel generated at this stage exhibits an increased degree of continuity and is hypothesized to integrate with the remaining N-A-S-H gel, potentially forming a hybrid gel structure. This structural evolution enhances the connectivity of the binding phase and promotes the development of a more coherent matrix. As a result, the overall structural integrity is partially restored, giving rise to a noticeable recovery in compressive strength. When the CaO dosage is further increased to 10% (GEO-5), although the liquid-to-solid ratio continues to rise and the formation of C-(A)-S-H gel is further promoted, the structural integrity of the system does not continue to improve. Instead, additional degradation mechanisms are introduced. On the one hand, excessive Ca(OH)_2_ undergoes extensive carbonation at later stages, forming CaCO_3_. While these carbonate phases may locally fill pores, the crystallization process may cause volumetric changes that could potentially induce microcracks within the matrix, thereby disrupting structural continuity. On the other hand, the excessive availability of Ca^2+^ leads to an imbalance in gel composition, low-polymerized C-(A)-S-H gel becomes dominant, whereas the highly polymerized N-A-S-H network is further suppressed. This results in a less cohesive and mechanically weaker framework. In addition, the increased liquid-to-solid ratio contributes to a higher volume of residual pores, and this effect, combined with carbonation-induced structural damage, ultimately leads to a renewed decrease in compressive strength. The possible limited reaction of CaO with the aluminosilicate network cannot be excluded, as suggested by the changes in NMR species and FTIR peak shifts. However, the extent of such reaction appears to be minor compared to the dominant hydration and carbonation processes, and its contribution to strength development is limited.

**Figure 7:**
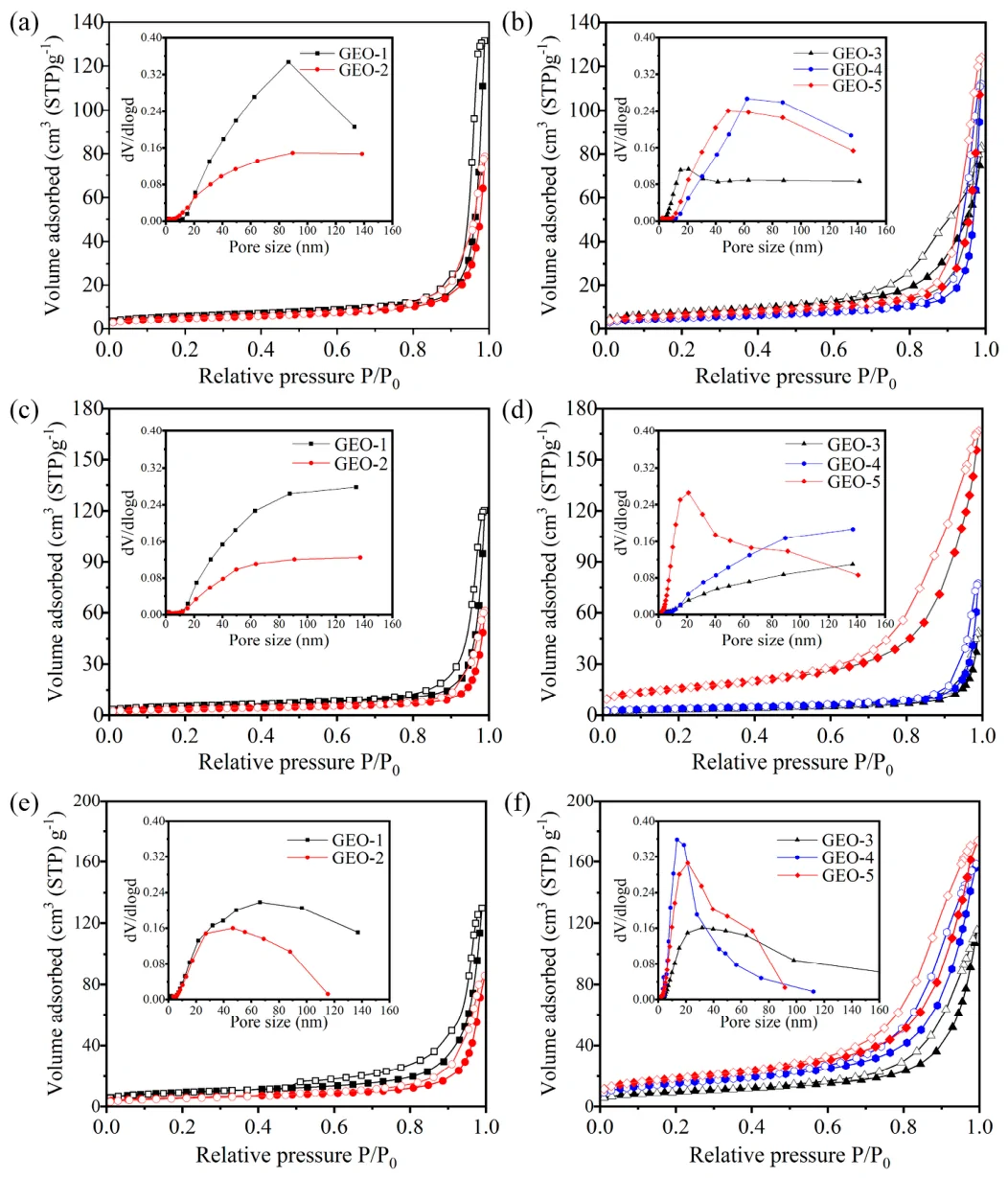
Nitrogen adsorption–desorption isotherms of (**a**) GEO-1 and GEO-2 at 3 d curing time; (**b**) GEO-3, GEO-4 and GEO-5 at 3 d curing time; (**c**) GEO-1 and GEO-2 at 7 d curing time; (**d**) GEO-3, GEO-4 and GEO-5 at 7 d curing time; (**e**) GEO-1 and GEO-2 at 28 d curing time; and (**f**) GEO-3, GEO-4 and GEO-5 at 28 d curing time.

In CaO-added geopolymers, calcium participates in the reaction through two primary pathways: it interacts with reactive silica to form C-(A)-S-H gel, and it hydrates to produce calcium hydroxide, which can subsequently carbonate into calcium carbonate. However, the compressive strength of geopolymer samples is closely related to their specific area and pore structure [33]. Therefore, the particle size of all geopolymer samples was strictly controlled at 1 mm, and the N_2_ adsorption and desorption isotherms were obtained and shown in Figure 7. The specific surface area, pore volume, and average pore diameter were calculated using the Brunauer–Emmett–Teller (BET) equation and the adsorption branch data (Table 1). The pore size distribution curves (inset of Figure 7) reveal that the mesopores of all samples are mainly distributed in the range of 20–100 nm, and with increasing CaO dosage, the pore size distribution gradually shifts toward smaller pore sizes. Although the pore structure parameters vary numerically with curing age, they exhibit consistent trends with increasing CaO dosage, which closely correspond to the evolution of compressive strength. When the curing time was 28 days, GEO-1 sample shows a specific surface area of 33.3 m^2^/g, a pore volume of 0.1970 cm^3^/g, and an average pore diameter of 32.10 nm, and with increasing CaO dosage, the specific surface areas of GEO-2 and GEO-3 became 21.0 and 34.7 m^2^/g, respectively. The pore volume first decreases to 0.1300 cm^3^/g and then increases to 0.1761 cm^3^/g, while the average pore diameter reduces to 33.61 and 23.83 nm. When the CaO dosage reaches 7.5% (GEO-4), the specific surface area increases markedly to 54.8 m^2^/g, the pore volume rises to 0.2424 cm^3^/g, and the average pore diameter further decreases to 19.23 nm. This phenomenon of increased specific surface area and elevated pore volume yet refined pore size was attributed to the filling effect of an appropriate amount of C-S-H gels on macropores, accompanied by the introduction of a large number of mesopores via the accumulation of nano-scale gel particles, which was fully consistent with the trend of obvious recovery in the compressive strength of GEO-4. However, for GEO-5, the specific surface area further increased to 66.8 m^2^/g, the pore volume rose to 0.2659 cm^3^/g, and the average pore diameter continued to decrease to 17.29 nm. At this stage, the excessive C-S-H gels and fine CaCO_3_ crystals formed via the carbonation of Ca(OH)_2_ failed to further optimize the pore structure. Instead, they induced internal microcracks in the matrix due to volume expansion, resulting in an abnormal increase in specific surface area as well as impaired matrix integrity, which ultimately caused a secondary decline in compressive strength. This series of pore structure evolutions was highly consistent with the fluctuation in Q^4^(2Al) content and the continuous increase in Q^4^(0Al) content obtained from ^29^Si NMR. It further confirmed that CaO primarily disrupted the aluminosilicate network and regulated the formation of N-A-S-H and C-S-H gels, thereby altering the pore structure and matrix integrity of the geopolymer and ultimately affecting its mechanical properties.

It should be noted that, in the present study, the water-to-binder ratio was adjusted with increasing CaO dosage to guarantee comparable paste workability. Consequently, the CaO content and water-to-binder ratio are coupled variables in this experimental design. The observed evolutions in mechanical properties and microstructures cannot be uniquely attributed to CaO modification alone, as variation in water content also alters the geopolymerization process, porosity development and final compressive strength. Experiments with a constant water-to-binder ratio and factorial-design analysis would help further disentangle the individual contributions of these two factors, which will be considered in our future work.

Furthermore, although XRD, ^29^Si MAS NMR and N_2_ adsorption are widely accepted techniques in geopolymer research for inferring phase assemblage, silicate network connectivity and pore-scale features, they provide only indirect information and cannot offer direct proof for hybrid-gel cross-linking or carbonation-triggered microcrack development. The formation of X-ray amorphous C-(A)-S-H phases is inferred from the combination of FTIR and NMR results, rather than directly observed. Microcrack formation is merely postulated based on pore structure trends. Direct evidence from SEM/BSE-EDS, ^27^Al NMR or microcrack characterization would be required to further validate these hypothetical mechanisms, which remain targets for follow-up investigations.

## 3. Experimental

### 3.1. Materials

The chemical reagents used in this study included sodium hydroxide (98% purity) and calcium oxide (≥97% purity), all of which were purchased from Sinopharm Chemical Reagent Co., Ltd. (Shanghai, China). Metakaolin and silica fume were used as the main aluminosilicate precursors for the preparation of one-part geopolymer. The metakaolin was obtained by calcining kaolin at 700 °C for 12 h in a muffle furnace. The chemical composition and particle size distribution of metakaolin and silica fume were consistent with those reported in our previous work [14] and thus are not repeated here for brevity. It should be noted that, while the precursor preparation followed the same procedure as in previous work, the present study focuses on the effect of CaO as an additive, which has not been systematically investigated in one-part metakaolin-based geopolymers.

### 3.2. Preparation of One-Part Geopolymer

The solid alkali activator was prepared by a high-temperature melting method according to our previous study [14]. Sodium hydroxide and quartz powder were uniformly mixed in a mass ratio of 1:1, ground in a planetary ball mill (YXQM-2L, MITR, Changsha, China) at 500 rpm for 10 min, and then transferred to a muffle furnace (KSL-1200X, Kejing, Hefei, China) for calcination at 650 °C for 4 h. After naturally cooling to room temperature, the product was ground to pass completely through a 75 μm standard sieve and then sealed and stored for later use. The total mass of cementitious materials was fixed at 100 g, and calcium oxide was introduced by equal mass replacement of the total cementitious materials with dosages of 0, 2.5, 5.0, 7.5 and 10.0%, respectively. The proportions of metakaolin, silica fume, and solid activator were adopted from our previous work [14], in which the Si/Al molar ratio of the system was optimized to approximately 2.0 to favor geopolymerization. For all mixtures, CaO was introduced as an external modifier, while the aluminosilicate precursor proportions remained unchanged. As shown in Table 2, the CaO dosages were selected to cover a range from 0 to 10 wt% at intervals of 2.5 wt%, enabling a systematic evaluation of its effect on the geopolymer properties. The water-to-binder ratio was gradually increased from 0.400 to 0.500 with increasing CaO content to ensure similar paste workability, as the water demand of the paste increases with CaO addition due to its rapid hydration [15].

The weighed solid alkali activator, metakaolin, silica fume and calcium oxide were poured into a mixing pot and dry-mixed for 2 min to achieve uniform mixing. Then, deionized water was added according to the preset water-to-binder ratio, and the mixture was rapidly stirred for 5 min to form a uniform paste with good fluidity. The paste was cast into 40 mm × 40 mm × 40 mm cubic molds and placed on a vibrating table for 5 min to eliminate internal bubbles. The samples were demolded after sealed curing for 24 h at room temperature and continued to be cured under the same conditions until the ages of 3, 7 and 28 d for mechanical property tests and microstructural characterizations, respectively.

### 3.3. Characterization

The uniaxial compressive strength of geopolymer samples was tested using a universal testing machine (CSS-55500, SINOTEST, Changchun, China). The chemical compositions of metakaolin, silica fume and solid activator were determined by an X-ray fluorescence spectrometer (XRF, Zetium, PANalytical, Almelo, The Netherlands). X-ray diffraction (XRD, Bruker D8 Advance, Karlsruhe, Germany) was employed to identify the phase composition of the samples using Cu Kα radiation at a scanning speed of 2°/min over a 2θ range of 5–70°. The chemical functional groups and chemical bond changes of the samples were characterized by Fourier transform infrared spectroscopy (FTIR, IRTracer 100, Shimadzu, Kyoto, Japan). The thermal weight loss behavior of the samples was investigated by a simultaneous thermal analyzer (TG-DTG, STA 449 F5, Netzsch, Selb, Germany) under a nitrogen atmosphere with a heating rate of 10 °C/min. The coordination environments of ^29^Si in the samples were analyzed by a solid-state nuclear magnetic resonance spectrometer (NMR, JEOL JNM-ECZL600G, JEOL, Akishima, Japan). The specific surface area, pore volume and pore size distribution of the samples were measured by a specific surface area and pore size analyzer (BET, BELSORP Max II, MicrotracBEL, Osaka, Japan).

## 4. Conclusions

In the present study, the effect of CaO additive on the compressive strength and microstructure of metakaolin-based one-part geopolymers was systematically investigated. The sample without CaO additive achieved the highest compressive strength of 56.6 MPa at 28 days. However, the sample with the addition of CaO showed the 28-day strengths lower than 25 MPa. XRD, FTIR and TG results indicated that CaO rapidly hydrated to form Ca(OH)_2_, which was continuously carbonated to calcite. ^29^Si NMR results suggested that the content of Q^4^(2Al), the characteristic structural unit of N-A-S-H gel, decreased firstly and then to some extent increased, while the content of Q^4^(0Al) increased monotonically, implying that Ca^2+^ preferentially consumed reactive silicon and potentially depolymerized the aluminosilicate network, which is the fundamental cause of the strength deterioration of geopolymers. At a moderate CaO dosage of 7.5%, the hydration and carbonation products could fill the pores and refine the pore size, corresponding to the strength rebound of the sample. At an excessive CaO dosage of 10%, the volume expansion of carbonation products may induce microcracks in the matrix and impair the structural integrity, which ultimately led to the secondary decline in strength. Therefore, the adverse effects of CaO on metakaolin-based one-part geopolymers originated from its rapid hydration, strong carbonation tendency, and the preferential consumption of reactive silicon by Ca^2+^, which may lead to the partial substitution of the high-polymerization-degree N-A-S-H network by low-polymerization-degree C-S-H gel and calcium carbonate. Only at a moderate CaO dosage of 7.5% can partial strength recovery be achieved through the hypothesized crosslinking of mixed gels and pore filling via carbonation products.

## Figures and Tables

**Table 1:** Specific surface area, pore volume, and pore size of geopolymer samples.

Curing Age	Sample	SBET (m^2^/g)	Pore Volume (cm^3^/g)	Pore Size (nm)
3 d	GEO-1	21.1	0.2017	48.9149
	GEO-2	16.9	0.1203	34.3494
	GEO-3	26.9	0.1275	21.0164
	GEO-4	18.0	0.1722	44.5851
	GEO-5	23.1	0.1913	20.9694
7 d	GEO-1	19.5	0.1843	48.1361
	GEO-2	12.8	0.0932	38.1663
	GEO-3	11.6	0.0746	31.619
	GEO-4	14.0	0.1187	40.3427
	GEO-5	57.3	0.2561	18.9832
28 d	GEO-1	33.3	0.1970	32.1023
	GEO-2	21.0	0.1300	33.6109
	GEO-3	34.7	0.1761	23.8306
	GEO-4	54.8	0.2424	19.2348
	GEO-5	66.8	0.2659	17.2876

**Table 2:** Mixture formulations for geopolymer preparation.

Sample	Solid Activator/g	Metakaolin/g	Silica Fume/g	CaO/g	Liquid/Solid Ratio
GEO-1	20.00	51.59	28.41	0.00	0.400
GEO-2	19.50	50.30	27.70	2.50	0.425
GEO-3	19.00	49.01	26.99	5.00	0.450
GEO-4	18.50	47.72	26.28	7.50	0.475
GEO-5	18.00	46.43	25.57	10.00	0.500

## Data Availability

The original contributions presented in this study are included in the article. Further inquiries can be directed to the corresponding authors.
